# The influence of sex on lower limb biomechanical characteristics during single-leg drop landing in individuals with unilateral functional ankle instability

**DOI:** 10.3389/fbioe.2025.1703251

**Published:** 2025-10-15

**Authors:** Huizi Cui, Zilong Wang, Minjie Lin, Tao Liu, Xiangdong Wang

**Affiliations:** School of Physical Education, Jimei University, Xiamen, China

**Keywords:** functional ankle instability, sex, single-leg drop landing, lower limb biomechanics, compensatory strategies

## Abstract

**Objective:**

To investigate sex differences in lower limb biomechanics during single-leg drop landing in individuals with unilateral Functional Ankle Instability (FAI).

**Methods:**

Twenty individuals with unilateral FAI (10 males/10 females) performed single-leg drop landings on unstable and stable limbs respectively. Kinematic data were captured using a Vicon motion capture system, and kinetic data were collected using force plates. Data were analyzed using a 2 × 2 (Sex × Side) mixed-design ANOVA.

**Results:**

Significant main effects of Sex were observed for hip flexion and abduction angles at both IC and peak vGRF, with females showing greater hip flexion (*p* = 0.005; *p* = 0.023) and smaller hip abduction (*p* = 0.026; *p* = 0.003) than males. A significant main effect of Sex was also found for ankle inversion at IC, with females exhibiting greater inversion than males (*p* = 0.005). For ankle plantarflexion at IC, a significant Sex × Side interaction was detected (*p* = 0.020); simple effects analysis revealed that females had reduced plantarflexion on the unstable side compared to their stable side (*p* = 0.010). For peak vGRF, a significant Sex × Side interaction was observed (*p* = 0.013); *post hoc* tests indicated that females had higher peak vGRF on the unstable side than males (*p* = 0.008) and their own stable side (*p* < 0.001). No significant differences were observed for knee joint angles, T_vGRF, LR, ΔL, or K_leg_ (*p* > 0.05).

**Conclusion:**

Female individuals with FAI employ a hip-dominant compensatory strategy (increased flexion/reduced abduction) and exhibit greater ankle inversion than males. On the unstable side, they demonstrate reduced plantarflexion compared to their stable side. Males with FAI primarily adopt a joint stiffening strategy with restricted motion. Future research on FAI should incorporate sex differentiation in biomechanical assessments to develop targeted rehabilitation.

## 1 Introduction

The ankle joint, serving as a critical nexus for weight-bearing and dynamic stability within the human kinetic chain, ranks among the most frequently injured joints in athletic activities (Medina McKeon and Hoch, 2019; Doherty et al., 2016; Yu et al., 2022). Substantial research indicates that approximately 40% of individuals sustaining an ankle sprain may progress to Chronic Ankle Instability (CAI) if prompt and efficacious treatment and management are not instituted (Hershkovich et al., 2015). Functional Ankle Instability (FAI), constituting a core subtype of CAI, is primarily characterized by diminished joint stability, proprioceptive deficits, and impaired neuromuscular control (Wenning and Schmal, 2023; Meng et al., 2025). Although individuals with FAI typically lack overt structural damage, recurrent “giving-way” episodes profoundly compromise athletic performance and quality of life, concomitant with attenuated dynamic postural control and an elevated risk of re-injury (Liu et al., 2024). This impairment in dynamic postural control may necessitate that the central nervous system of individuals with FAI increase reliance on proximal joints to compensate for deficient proprioceptive input from the ankle. However, such compensatory mechanisms may exacerbate aberrant loading patterns in the affected lower limb and potentially induce compensatory strategies in the contralateral limb, thereby precipitating bilateral kinetic chain imbalances and establishing a vicious cycle of recurrent injury (Wang et al., 2024a).

Landing constitutes a ubiquitous maneuver in high-intensity sports such as basketball, volleyball, and soccer, and represents a quintessential high-risk scenario for ankle injuries, particularly re-injury among individuals with FAI (Meng et al., 2022; Han et al., 2022; Yu et al., 2024). The substantial impact forces generated instantaneously demand precise inter-joint coordination throughout the lower limb to effectively attenuate load and maintain stability. The biomechanical characteristics of the lower limb during landing directly dictate load distribution and injury risk across the ankle joint and the entire lower extremity kinetic chain (Wang et al., 2024b; Wang et al., 2024c). Previous studies on landing in individuals with FAI have revealed altered movement patterns and compensatory strategies, but a comprehensive understanding of these biomechanical adaptations remains limited, highlighting the need for further investigation.

Sex differences represent a pivotal and inescapable factor in sports biomechanics research (Li et al., 2025). Systematic disparities exist between males and females concerning hormonal profiles, anatomical structure, and neuromuscular control strategies (Alizadeh et al., 2023; Guo et al., 2022). Specifically, at the knee joint, sex differences in injury risk, such as anterior cruciate ligament injuries, are well-documented and often attributed to factors like lower limb alignment, relative hamstring weakness, and hormonal fluctuations (e.g., estrogen) that affect ligament laxity (Gilmer et al., 2025). Similarly, hip joint biomechanics during dynamic tasks exhibit sexual dimorphisms; for instance, females tend to demonstrate greater hip adduction and reduced hip abductor strength, which may influence proximal stability and compensatory mechanisms (Bedo et al., 2022). These fundamental biological distinctions may lead to divergent compensatory strategies and movement patterns in male and female individuals with FAI during landing impact. For instance, males, potentially leveraging superior muscle stiffness and strength, may exhibit a propensity towards a “stiffening” strategy; whereas females, potentially due to greater ligamentous laxity and differences in neuromuscular control patterns, may exhibit greater reliance on dynamic adjustments of proximal joints or manifest specific biomechanical vulnerabilities (Elshorbagy et al., 2024; Zhang et al., 2025; Jonasson et al., 2023). Nevertheless, existing laboratory investigations involving individuals with FAI frequently neglect to adequately account for sex-related influences. Crucially, dedicated research examining sex-specific lower limb biomechanical characteristics during single-leg drop landing tasks in individuals with unilateral FAI remains conspicuously absent. Concurrently, the literature predominantly focuses on statistically significant differences while overlooking the potential value of non-significant findings. As emphasized by Abadie (2024), “findings of no effect are equally foundational for theory development, necessitating explicit reporting and discussion of non-significant results.” Consequently, comprehensively elucidating the influence of sex differences on lower limb biomechanics in individuals with FAI holds significant import. Such investigation would not only deepen our understanding of sex-specific biomechanical manifestations of FAI but also provide crucial insights for clinically developing individualized rehabilitation protocols.

Therefore, this study was aimed to employ biomechanical analysis to characterize the lower limb biomechanics of male and female individuals with unilateral FAI during single-leg drop landing. Based on prior research, the following hypotheses are proposed: 1) Male and female individuals with FAI will employ distinct lower limb biomechanical strategies during single-leg drop landing; 2) The unstable limb will exhibit sex-specific biomechanical patterns, with female unstable limbs demonstrating a heightened propensity towards forming high-risk loading mechanisms.

## 2 Methods

### 2.1 Participants

The requisite sample size was estimated *a priori* utilizing G*Power software (version 3.1.9.7), informed by previous relevant studies (Wang et al., 2024a; Ma et al., 2025) and the planned statistical analyses. Statistical power (1-β), Type I error rate (α), and effect size (f) were set at 0.8, 0.05, and 0.4, respectively, yielding a minimum sample size of 16 participants. In accordance with the International Ankle Consortium criteria (Gribble et al., 2014), 20 participants with unilateral FAI (10 males, 10 females; possessing comparable athletic backgrounds and physical characteristics) were recruited from the School of Physical Education, Soochow University. Inclusion was based on Cumberland Ankle Instability Tool (CAIT) scores, a self-reported questionnaire. The clinical examination included physical tests such as the anterior drawer test and talar tilt test to assess mechanical instability, ensuring that participants met the International Ankle Consortium criteria for FAI. In this study, all participants had unilateral FAI on their dominant limb. The dominant limb was determined via a kicking test for limb dominance (Ren et al., 2021). All assessments and screening procedures were administered by an experienced rehabilitation therapist. Prior to testing, all participants were fully apprised of the experimental procedures and provided written informed consent. Ethical approval was granted by the Soochow University Ethics Committee (Approval No: SUDA20241209H14). Participant demographics are presented in Table 1.

**TABLE 1:** Participant characteristics (M ± SD).

Characteristic	Male group (n = 10)	Female group (n = 10)
Age (year)	22.4 ± 1.3	22.5 ± 1.3
Height (cm)	178.8 ± 3.5	165.2 ± 4.0^*^
Weight (kg)	75.4 ± 5.7	62.4 ± 5.3^*^
BMI (kg/m2)	23.6 ± 1.3	22.9 ± 1.8
CAIT Score	17.1 ± 2.0	18.2 ± 2.1
Unstable Side (L/R)	(2/8)	(1/9)

*p < 0.05, similarly hereinafter.

Inclusion and exclusion criteria for the FAI group, consistent with prior research (Wang et al., 2024a; Meng et al., 2022; Wang et al., 2024b; Wang et al., 2024c), were as follows:

Inclusion Criteria: 1) History of at least one significant unilateral ankle sprain, with the initial injury occurring ≥12 months prior to testing and accompanied by inflammatory sequelae (e.g., pain, swelling) resulting in ≥1 day of interrupted physical activity; 2) History of “giving-way,” and/or recurrent sprains, and/or perceived instability on the affected ankle; 3) CAIT score ≤24; 4) FAI symptoms confined to one ankle; 5) Engagement in physical exercise ≥3 times per week.

Exclusion Criteria: 1) History of acute ankle sprain, surgery, or fracture; 2) Acute musculoskeletal injury to other lower limb joints within 1 month prior to testing; 3) Bilateral FAI; 4) Congenital joint deformities; 5) Positive talar tilt or anterior drawer test indicative of mechanical instability; 6) CAIT score >24; 7) Female participants undergoing menses on the day of testing.

### 2.2 Instrumentation

#### 2.2.1 Vicon Infrared 3D motion capture system

The system comprised 8 infrared cameras (Model: MX13, United Kingdom), MX Net, MX Control, PC workstation, calibration kit, and standard accessories. Data were captured at 100 Hz. The Vicon CGM 2.3 lower body model was implemented.

#### 2.2.2 Three-dimensional force plates

Two Kistler force plates (90 cm × 60 cm × 10 cm, Model: 9287B, Switzerland) were embedded flush with the laboratory floor at the center of the motion capture volume. Force plate data were sampled at 1,000 Hz. Synchronization between the Kistler force plates and the Vicon motion capture system was achieved via an analog-to-digital converter.

### 2.3 Experimental design and procedures

**FIGURE 1:**
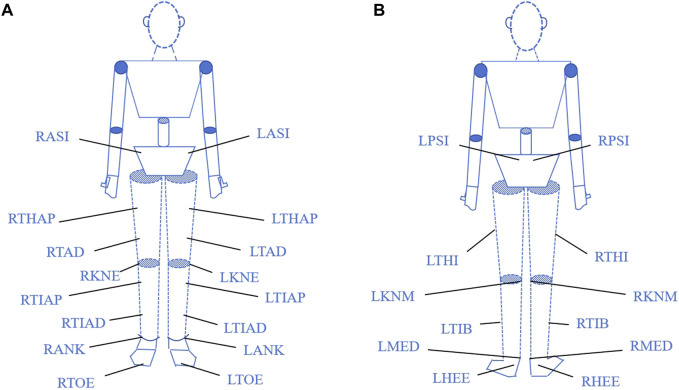
Marker Placement Schematic **(A)** Anterior View; **(B)** Posterior View. Note: Adapted from Wang et al. (2024b). LANK: Left lateral malleolus; LASI: Left anterior superior iliac spine; LHEE: Left heel; LKNM: Left medial knee; LKNE: Left lateral knee; LMED: Left medial malleolus; LPSI: Left posterior superior iliac spine; LTHAP: Left thigh proximal; LTHI: Left thigh; LTAD: Left thigh distal; LTIB: Left tibia; LTIAD: Left tibia distal; LTIAP: Left tibia proximal; LTOE: Left toe; RANK: Right lateral malleolus; RASI: Right anterior superior iliac spine; RHEE: Right heel; RKNM: Right medial knee; RKNE: Right lateral knee; RMED: Right medial malleolus; RPSI: Right posterior superior iliac spine; RTHAP: Right thigh proximal; RTHI: Right thigh; RTAD: Right thigh distal; RTIB: Right tibia; RTIAD: Right tibia distal; RTIAP: Right tibia proximal; RTOE: Right toe.

Prior to each testing session, participants wore standardized laboratory apparel (compression shorts and athletic shoes), which was sized for individual fit to ensure comfort. Following a standardized warm-up, 28 retroreflective markers (14 mm diameter) were affixed to anatomical landmarks according to the CGM 2.3 lower body model protocol (Figure 1) (Wang et al., 2024b). To ensure consistency, all marker placement was performed by the same experienced technician.

### 2.4 Single-leg drop landing protocol

**FIGURE 2:**
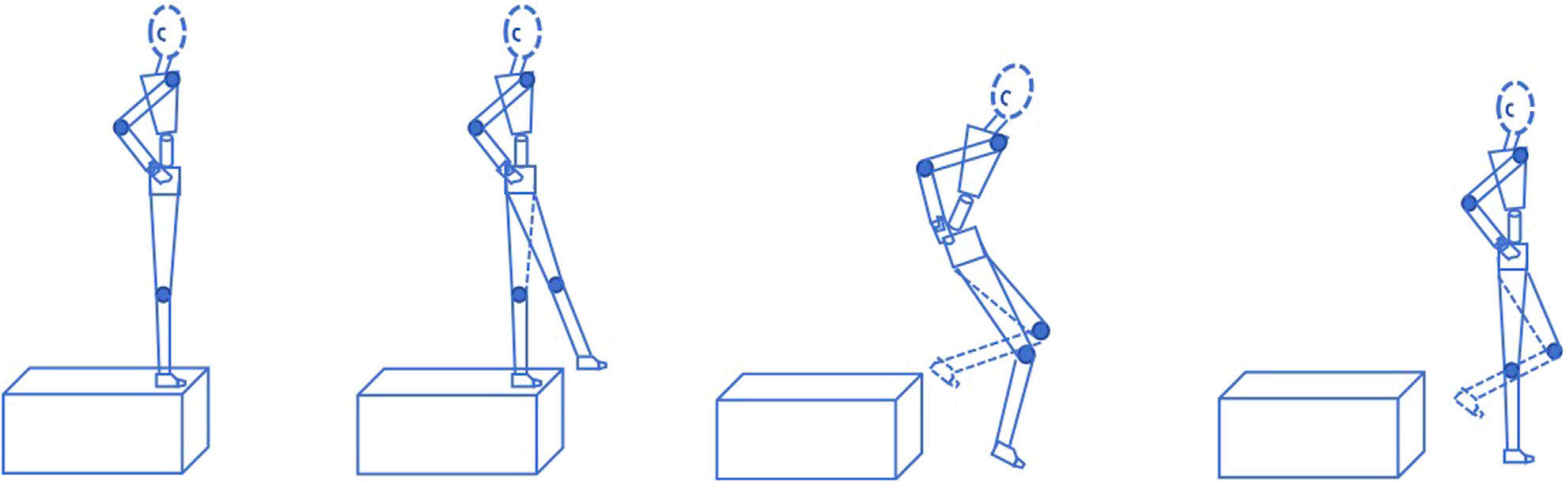
Schematic of single-leg drop landing task.

The single-leg drop landing task was adapted from a protocol previously described by Wang et al. (2024c). Participants performed a “toe-to-heel” single-leg drop landing (Figure 2), maintaining hands on hips to minimize arm swing. Prior to formal testing, all participants were given sufficient practice trials to familiarize themselves with and master the landing maneuver. A trial was discarded and repeated if the participant (a) the contralateral limb contacted the ground or force plate, (b) any hop, step, or arm abduction occurred after landing, or (c) hands left the hips. The testing order for the stable and unstable sides was alternated. To mitigate random error and enhance data reliability, participants performed three successful trials for each landing condition (unstable limb landing, stable limb landing). The average of the three trials was used for subsequent analysis.

### 2.5 Data processing and analysis

Raw kinematic data from the Vicon system and kinetic data from the force plates were processed and analyzed using Visual 3D software (C-Motion, Inc., United States). Three-dimensional coordinate data were smoothed using a 4th order low-pass Butterworth filter with a cutoff frequency of 10 Hz. Force plate data were filtered using an identical filter with a 50 Hz cutoff frequency. Joint angles for the lower extremities were calculated using an X-Y-Z Cardan rotation sequence (Euler angles). Initial contact (IC) was defined as the instant when the vertical ground reaction force (vGRF) first exceeded 10 N (Wang et al., 2024b; Wang et al., 2024c).

Given that injury vulnerability in individuals with FAI during landing is heightened at IC, the instant of peak vGRF, and when the ankle is positioned in plantarflexion and inversion (Watabe et al., 2024; Han et al., 2023), the primary outcome measures for this study were:

1. Hip, knee, and ankle joint angles (°) in the sagittal and frontal planes at IC and at the time of peak vGRF.
2. Peak vGRF, normalized to body weight (BW).
3. Time to peak vGRF (T_vGRF, ms).
4. Vertical loading rate (LR, BW/ms), calculated as: LR = Peak vGRF/T_vGRF.
5. Change in lower limb length (ΔL, m), defined as the displacement of the greater trochanter marker from its position at IC to its lowest position during the landing phase.
6. Lower limb stiffness (K_leg_, BW/m), calculated as: K_leg_ = Peak vGRF/ΔL.

### 2.6 Statistical analysis

Statistical analyses were performed using SPSS software (Version 27.0, IBM Corp., United States). Descriptive statistics are presented as means ± standard deviations (M ± SD). The normality of data distribution was assessed using the Shapiro-Wilk test, and homogeneity of variance was evaluated using Levene’s test. A 2 (Sex: Male, Female) × 2 (Side: Unstable, Stable) mixed-design analysis of variance (ANOVA) was employed to analyze the kinematic and kinetic variables. Mauchly’s Test of Sphericity was utilized to assess the assumption of sphericity; where this assumption was violated, Greenhouse-Geisser corrections were applied to adjust the degrees of freedom and F-values. Statistical significance was set at α = 0.05. In instances where significant interaction effects were detected, simple main effects analyses were conducted. Bonferroni correction was applied for multiple pairwise comparisons within simple effects, resulting in an adjusted significance level of p < 0.0125.

## 3 Results

### 3.1 Hip joint angle characteristics in individuals with FAI

Significant main effects of Sex were observed for hip flexion and hip abduction angles at both IC and the time of peak vGRF. Female individuals with FAI demonstrated significantly greater hip flexion angles and smaller hip abduction angles compared to male participants at both time points (*p* = 0.005; *p* = 0.026; *p* = 0.023; *p* = 0.003) (Table 2).

**TABLE 2:** Hip Joint Angles During Single-Leg Drop Landing at IC and Peak vGRF.

Variable	Unstable side	Stable side	Main effect		Interaction effect
			Sex	Side	Sex × Side
IC					
Hip Flexion (+)/Extension (−) (°)					
Male	17.47 ± 5.58	19.57 ± 4.80	***p* = 0.005** ^*^	*p* = 0.239	*p* = 0.612
			*F* = 10.176	*F* = 1.486	*F* = 0.266
Female	23.91 ± 4.39	24.76 ± 4.71	*Eta* ^*2*^ = 0.361	*Eta* ^*2*^ = 0.076	*Eta* ^*2*^ = 0.015
Hip Adduction (+)/Abduction (−) (°)					
Male	−5.61 ± 2.53	−5.56 ± 2.81	***p* = 0.026** ^*^	*p* = 0.850	*p* = 0.902
			*F* = 5.867	*F* = 0.037	*F* = 0.016
Female	−3.51 ± 2.97	−3.26 ± 2.31	*Eta* ^*2*^ = 0.246	*Eta* ^*2*^ = 0.002	*Eta* ^*2*^ = 0.001
Peak vGRF					
Hip Flexion (+)/Extension (−) (°)					
Male	22.91 ± 7.56	23.32 ± 6.91	***p* = 0.023** ^*^	*p* = 0.522	*p* = 0.694
			*F* = 6.131	*F* = 0.426	*F* = 0.159
Female	27.82 ± 4.42	29.51 ± 5.29	*Eta* ^*2*^ = 0.254	*Eta* ^*2*^ = 0.023	*Eta* ^*2*^ = 0.009
Hip Adduction (+)/Abduction (−) (°)					
Male	−5.36 ± 2.96	−5.24 ± 2.44	***p* = 0.003** ^*^	*p* = 0.944	*p* = 0.797
			*F* = 11.444	*F* = 0.005	*F* = 0.068
Female	−2.33 ± 2.12	−2.55 ± 1.90	*Eta* ^*2*^ = 0.389	*Eta* ^*2*^ < 0.001	*Eta* ^*2*^ = 0.004

The bolded text indicates that this indicator exhibits a statistically significant difference, similarly hereinafter.

### 3.2 Knee joint angle characteristics in individuals with FAI

No statistically significant differences (*p* > 0.05) were observed for any knee joint angle measures (flexion/extension, varus/valgus) at either IC or the time of peak vGRF across Sex, Side, or their interaction (Table 3).

**TABLE 3:** Knee Joint Angles During Single-Leg Drop Landing at IC and Peak vGRF.

Variable	Unstable side	Stable side	Main effect		Interaction effect
			Sex	Side	Sex × Side
IC					
Knee Flexion (+)/Extension (−) (°)					
Male	7.78 ± 3.25	8.61 ± 4.35	*p* = 0.933	*p* = 0.137	*p* = 0.126
			*F* = 0.007	*F* = 2.413	*F* = 2.562
Female	8.26 ± 3.17	8.99 ± 4.72	*Eta* ^*2*^ < 0.001	*Eta* ^*2*^ = 0.113	*Eta* ^*2*^ = 0.119
Knee Varus (+)/Valgus (−) (°)					
Male	1.70 ± 2.98	2.08 ± 1.93	*p* = 0.648	*p* = 0.746	*p* = 0.691
			*F* = 0.216	*F* = 0.108	*F* = 0.163
Female	1.48 ± 2.03	1.44 ± 2.41	*Eta* ^*2 =*^ 0.012	*Eta* ^*2*^ = 0.006	*Eta* ^*2*^ = 0.009
Peak vGRF					
Knee Flexion (+)/Extension (−) (°)					
Male	28.11 ± 5.29	28.24 ± 7.60	*p* = 0.419	*p* = 0.916	*p* = 0.989
			*F* = 0.685	*F* = 0.012	*F* < 0.001
Female	26.05 ± 5.54	26.21 ± 6.61	*Eta* ^*2*^ = 0.037	*Eta* ^*2*^ = 0.001	*Eta* ^*2*^ < 0.001
Knee Varus (+)/Valgus (−) (°)					
Male	1.37 ± 2.13	1.62 ± 2.51	*p* = 0.982	*p* = 0.921	*p* = 0.624
			*F* = 0.001	*F* = 0.010	*F* = 0.248
Female	1.70 ± 2.23	1.33 ± 2.40	*Eta* ^*2*^ < 0.001	*Eta* ^*2*^ = 0.001	*Eta* ^*2*^ = 0.014

### 3.3 Ankle joint angle characteristics in individuals with FAI

A significant Sex × Side interaction effect was observed for ankle plantarflexion angle at IC (*p* = 0.020). Simple effects analysis revealed that female individuals with FAI exhibited significantly reduced plantarflexion angles on their unstable side compared to their stable side (*p* = 0.010). A significant main effect of Sex was found for ankle inversion angle at IC (*p* = 0.005), with female individuals with FAI demonstrating greater inversion angles than males (Table 4).

**TABLE 4:** Ankle joint angles during single-leg drop landing at IC and Peak vGRF.

Variable	Unstable side	Stable side	Main effect		Interaction effect
			Sex	Side	Sex × Side
IC					
Ankle Dorsiflexion (+)/Plantarflexion (−) (°)					
Male	−21.77 ± 4.48	−20.76 ± 4.28	*p* = 0.438	*p* = 0.154	***p* = 0.020** ^*^
			*F* = 0.629	*F* = 2.220	*F* = 6.561
Female	−20.65 ± 3.47	−24.47 ± 4.56	*Eta* ^*2*^ = 0.034	*Eta* ^*2*^ = 0.110	*Eta* ^*2*^ = 0.267
Ankle Inversion (+)/Eversion (−) (°)					
Male	1.94 ± 2.77	2.33 ± 2.45	***p* = 0.005** ^*^	*p* = 0.355	*p* = 0.713
			*F* = 10.032	*F* = 0.900	*F* = 0.139
Female	4.72 ± 2.22	5.63 ± 3.03	*Eta* ^*2*^ = 0.358	*Eta* ^*2*^ = 0.048	*Eta* ^*2*^ = 0.008
Peak vGRF					
Ankle Dorsiflexion (+)/Plantarflexion (−) (°)					
Male	13.75 ± 3.15	16.04 ± 7.34	*p* = 0.127	*p* = 0.627	*p* = 0.292
			*F* = 2.564	*F* = 0.245	*F* = 1.180
Female	12.73 ± 3.88	11.87 ± 3.97	*Eta* ^*2*^ = 0.125	*Eta* ^*2*^ = 0.013	*Eta* ^*2*^ = 0.062
Ankle Inversion (+)/Eversion (−) (°)					
Male	2.40 ± 1.98	3.26 ± 2.37	*p* = 0.478	*p* = 0.567	*p* = 0.454
			*F* = 0.524	*F* = 0.339	*F* = 0.585
Female	2.32 ± 2.12	2.21 ± 2.55	*Eta* ^*2*^ = 0.028	*Eta* ^*2*^ = 0.019	*Eta* ^*2*^ = 0.031

The bolded text indicates that this indicator exhibits a statistically significant difference, similarly hereinafter.

### 3.4 Kinetic characteristics in individuals with FAI

A significant Sex × Side interaction effect was detected for peak vGRF (*p* = 0.013). Subsequent simple effects analysis revealed that on the unstable side, female individuals with FAI demonstrated significantly elevated peak vGRF values compared to their male counterparts (*p* = 0.008). Furthermore, female individuals with FAI exhibited significantly greater peak vGRF on their unstable side relative to their own stable side (*p* < 0.001) (Table 5).

**TABLE 5:** Peak vGRF, T_vGRF, LR, ΔL, and K_leg_ During Single-Leg Drop Landing.

Variable	Unstable side	Stable side	Main effect		Interaction effect
			Sex	Side	Sex × Side
Peak vGRF (BW)					
Male	2.51 ± 0.54	2.34 ± 0.53	***p* = 0.039** ^*^	***p* < 0.001** ^*^	***p* = 0.013** ^*^
			*F* = 4.953	*F* = 17.048	*F* = 7.674
Female	3.30 ± 0.63	2.41 ± 0.31	*Eta* ^*2*^ = 0.216	*Eta2* = 0.486	*Eta* ^*2*^ = 0.299
T_vGRF (ms)					
Male	62.00 ± 15.49	70.00 ± 17.64	*p* = 0.523	*p* = 0.809	*p* = 0.104
			*F* = 0.425	*F* = 0.060	*F* = 2.940
Female	73.00 ± 15.67	67.00 ± 17.03	*Eta* ^*2*^ = 0.023	*Eta* ^*2*^ = 0.003	*Eta* ^*2*^ = 0.140
LR (BW/ms)					
Male	0.42 ± 0.19	0.41 ± 0.16	*p* = 0.729	*p* = 0.559	*p* = 0.657
			*F* = 0.124	*F* = 0.355	*F* = 0.204
Female	0.45 ± 0.12	0.42 ± 0.11	*Eta* ^*2*^ = 0.007	*Eta* ^*2*^ = 0.019	*Eta* ^*2*^ = 0.011
ΔL (m)					
Male	0.14 ± 0.03	0.12 ± 0.02	*p* = 0.289	*p* = 0.119	*p* = 0.151
			*F* = 1.192	*F* = 2.679	*F* = 2.252
Female	0.13 ± 0.03	0.12 ± 0.02	*Eta* ^*2*^ = 0.062	*Eta* ^*2*^ = 0.130	*Eta* ^*2*^ = 0.111
K_leg_ (BW/m)					
Male	21.65 ± 3.47	23.52 ± 3.41	*p* = 0.153	*p* = 0.194	*p* = 0.820
			*F* = 2.230	*F* = 1.824	*F* = 0.053
Female	23.37 ± 3.23	24.70 ± 3.56	*Eta* ^*2*^ = 0.110	*Eta* ^*2*^ = 0.092	*Eta* ^*2*^ = 0.003

The bolded text indicates that this indicator exhibits a statistically significant difference, similarly hereinafter.

## 4 Discussion

### 4.1 Influence of sex differences on lower limb movement strategies in FAI during landing

This investigation provides a biomechanical elucidation of sex-specific compensatory patterns adopted by individuals with unilateral FAI during single-leg drop landing. The results demonstrate that, relative to their male counterparts, female individuals with FAI exhibit significantly greater hip flexion and reduced hip abduction angles at both IC and the instant of peak vGRF, concomitant with elevated ankle inversion angles at IC. These findings robustly support Hypothesis 1, confirming that male and female individuals with FAI employ divergent lower limb biomechanical strategies to compensate for compromised ankle stability.

Specifically, female individuals with FAI appear to augment dynamic hip flexion control to offset ankle instability deficits, whereas male individuals with FAI likely leverage their inherently greater muscle stiffness to adopt a more “stiffening” support strategy. Previous research has documented that individuals with FAI increase reliance on hip flexion for impact attenuation during dynamic tasks as a consequence of ankle impairment (Lin et al., 2019; Son et al., 2019). The current results indicate that this compensatory behavior is markedly more pronounced in females, highlighting the critical role of sex differences in FAI compensatory mechanics. These findings underscore significant sex-based divergences in hip flexion compensation, highlighting the necessity of incorporating sex as a fundamental factor in FAI biomechanical research. These sexually dimorphic biomechanical adaptations are likely intrinsically linked to systematic differences between males and females in anatomical structure, hormonal milieu, and neuromuscular control paradigms.

From an anatomical perspective, females typically possess a wider pelvis, larger Q-angle, and exhibit greater ligamentous laxity mediated by estrogen. These characteristics may predispose females to rely more heavily on active control of hip abductor musculature—referring to neuromuscular activation and coordination of muscles like the gluteus medius—to maintain proximal stability during dynamic tasks. The observed reduced hip abduction angle in females with FAI may indicate suboptimal activation of these muscles. This compromised activation can compromise frontal plane stability and increase injury risk (Sugimoto et al., 2014; Jacobs et al., 2007), and is consistent with the known baseline tendency for reduced hip abduction in females (You et al., 2023; Liu et al., 2021). Furthermore, extant research indicates that testosterone in males promotes muscle fiber hypertrophy and increases the proportion of type II fast-twitch fibers, thereby significantly enhancing muscle stiffness and explosive power (Ahtiainen et al., 2003; Cadore et al., 2012). This physiological advantage may facilitate a male propensity towards “stiffening” compensation by reducing joint range of motion when encountering high-impact landing tasks. It is noteworthy that the genesis of these sex differences is not solely attributable to anatomical disparities but is also intricately linked to hormonal fluctuations and neuromuscular control strategies. Studies reveal that females exhibit heightened ligamentous laxity during physical activity, closely associated with cyclical fluctuations in endogenous estrogen levels (Mancino et al., 2024). This physiological trait may necessitate a greater reliance on conservative control strategies involving the hip abductors to maintain dynamic stability during high-impact landings in females. However, diminished hip abduction angles indirectly suggest suboptimal activation of the gluteus medius, potentially compromising frontal plane stability, increasing the risk of compensatory pelvic tilt, and precipitating kinetic chain imbalances (Sugimoto et al., 2014). This observation aligns with Hunter’s proposition that inherent physiological and anatomical sex differences constitute pivotal determinants of divergent neuromuscular performance between males and females (Hunter, 2014). Concurrently, research by Jacobs et al. (2007) demonstrated that females generate significantly lower peak hip abduction torque during landing compared to males, further underscoring the critical role of hip abductor function in female motor control. The findings of Gehring et al. (2014), indicating greater hip adduction angles in females during running tasks, lend additional support to the notion of significant sex differences in hip abduction control. The results of the present study corroborate prior conclusions regarding the influence of sex differences on movement execution, demonstrating that despite FAI-induced proprioceptive deficits and diminished neuromuscular control, sex-specific disparities in hip joint mechanics persist.

Despite observable sex differences in hip and ankle kinematics, no significant differences in knee joint angles were detected in this study. This phenomenon may be attributable to the unique biomechanical role of the knee. Appropriate modulation of knee flexion angle can effectively amplify impact attenuation during landing, thereby enhancing postural stability to compensate for reduced ankle stability (Tamura et al., 2017). Research by Blackburn and Padua suggests that a moderate increase in knee flexion angle facilitates effective absorption of impact energy during the landing support phase, enhancing buffering capacity and stability (Blackburn and Padua, 2008). However, the current findings indicate that male and female individuals with FAI employed statistically indistinguishable strategies regarding knee flexion and frontal plane (varus/valgus) angle adjustments during this high-impact task. This suggests potential similarity in knee joint control strategies between sexes under these specific experimental conditions. Nevertheless, this result cannot be extrapolated to imply that individuals with FAI knee biomechanics align with those of healthy controls; it solely indicates an absence of sex differentiation in knee compensatory strategies within the parameters of this specific task.

### 4.2 Sex-specific biomechanical adaptations of the unstable limb and injury risk

This study further revealed that female individuals with FAI exhibit unique biomechanical characteristics specifically within their unstable limb during the single-leg drop landing task. Specifically, the unstable ankle demonstrated reduced plantarflexion compared to the stable side, accompanied by elevated peak vGRF. Moreover, peak vGRF on the unstable limb was significantly higher in females than in males. This outcome validates Hypothesis 2, uncovering a distinct biomechanical vulnerability in female individuals with FAI during landing. Prior research established that ankle injury in FAI impairs proprioception and neuromuscular control, detrimentally affecting muscle reaction timing and coordination, and diminishing the perception of joint position and motion (Yokoyama et al., 2008; Xue et al., 2021; Kang et al., 2022). The present findings accentuate the manifestation of this deficit in female individuals with FAI, suggesting they may adopt a more conservative movement strategy to mitigate loading on the compromised ankle. Paradoxically, the results indicate that this conservative approach failed to effectively alleviate aberrant loading on the unstable limb. Insufficient ankle plantarflexion at IC may alter the foot-ground interaction, potentially reducing the time available for eccentric energy absorption by the plantarflexors. Since the subsequent transition into dorsiflexion is critical for attenuating impact forces during landing (Yeow et al., 2011), the reduced plantarflexion observed in females with FAI may compromise this energy-absorbing phase. This may compromise the eccentric energy-absorbing capacity of the gastrocnemius-soleus complex and potentially facilitate the transmission of impact forces proximally. This interpretation is consistent with the observed greater hip flexion and reduced hip abduction angles in female individuals with FAI compared to males.

From a neurophysiological perspective, analysis of brain connectivity in 428 males and 521 females by Ingalhalikar et al. (2013) revealed significantly stronger intrahemispheric connectivity in supratentorial regions among males, whereas females exhibited more pronounced interhemispheric connectivity. This suggests superior efficiency in motor coordination for males, while females may possess advantages in linguistic-emotional expression and perceptual processing. These divergent brain connectivity patterns may influence the selection of movement strategies by individuals with FAI. Female individuals with FAI, potentially leveraging their perceptual/emotional processing strengths, may exhibit a greater propensity towards conservative strategies to avoid injury; whereas males, benefiting from efficient motor coordination, may achieve more uniform load distribution through muscular stiffening. This may represent a deeper neurophysiological factor contributing to sex-differentiated motor control strategies, although the precise linkage to specific biomechanical manifestations during landing impact necessitates direct verification in future studies. Further analysis suggests that the underlying mechanism of this maladaptive biomechanics may also reside in a disrupted proprioceptive-motor control loop in FAI (Wang et al., 2024b). Diminished proprioceptive input from the unstable ankle may impair the effective recruitment of lower limb musculature for energy absorption, leading to focal impact loading on the unstable limb (Feger et al., 2014), a deleterious state significantly amplified in female individuals with FAI. Although the proximal compensatory strategy (enhanced hip flexion) observed in females may confer short-term postural stability, it may induce detrimental cascading effects. The hip, knee, and ankle function as interdependent components of the lower extremity kinetic chain. Dysfunction in one joint often precipitates altered movement patterns, engaging adjacent or proximal joints to compensate for the impaired segment (Li et al., 2019; Lee et al., 2018; Stanley et al., 2019). While such compensatory mechanisms may facilitate task completion acutely, chronic utilization risks disrupting kinetic chain coordination and elevating re-injury susceptibility. Furthermore, the absence of significant differences in T_vGRF, LR, and K_leg_ indicates that despite sex disparities in peak vGRF and ankle angles, male and female individuals with FAI may employ comparable control strategies concerning the rate of force development and overall lower limb deformation/stiffness during impact absorption.

In summary, rehabilitation for individuals with FAI must incorporate sex-specific strategies informed by these biomechanical distinctions. Specifically, for female individuals with FAI, rehabilitation protocols should prioritize enhancing eccentric plantarflexor control and facilitating gluteus medius activation. Such targeted training aims to rectify aberrant landing biomechanics and disrupt the detrimental compensatory cycle, thereby mitigating re-injury risk. For male individuals with FAI, the rehabilitation focus should shift towards optimizing energy transfer efficiency within their inherent stiffening strategy. Acknowledging their typically greater muscle strength and stiffness, interventions may incorporate elements like plyometric and power training to enhance efficiency. However, particular vigilance should be exercised to mitigate potential focal stress concentration risks (e.g., at the Achilles tendon) associated with over-reliance on muscular stiffening. Moreover, based on the present findings, it is strongly recommended that future biomechanical investigations of FAI explicitly stratify analyses by sex. This approach is essential for precisely delineating sex-specific biomechanical mechanisms underlying FAI, thereby enabling the formulation of highly targeted clinical rehabilitation guidelines.

### 4.3 Limitations

1) The relatively modest sample size and high homogeneity within the participant cohort may constrain statistical power and limit the generalizability of findings to the broader FAI population. 2) The standardized 30 cm single-leg drop landing task employing a prescribed “toe-to-heel” pattern, while ensuring experimental control, diverges from the complex and variable demands of real-world athletic scenarios, potentially impacting ecological validity. 3) The absence of synchronized surface electromyography (sEMG) data precludes definitive mechanistic interpretation of the observed kinematic and kinetic sex differences in terms of underlying neuromuscular control. Future investigations should endeavor to recruit larger, more diverse samples, simulate ecologically valid sport-specific landing scenarios, and integrate sEMG to comprehensively explore the sex-specific biomechanical signatures of FAI. Additionally, future studies should incorporate measurements of ankle joint moments, normalized to body weight, to better quantify joint loading in relation to sex differences and body size variations.

## 5 Conclusion

This study elucidated the influence of sex differences on lower limb biomechanics during single-leg drop landing in individuals with unilateral FAI. Female individuals with FAI exhibited a distinct biomechanical profile characterized by greater ankle inversion than males and reduced plantarflexion on the unstable limb compared to their stable side, along with elevated peak vGRF on the unstable limb. These findings indicate heightened vulnerability to dynamic instability and re-injury. Furthermore, divergent compensatory strategies were identified: Female individuals with FAI manifested a proximal (hip-dominant) compensatory tendency, evidenced by greater hip flexion and reduced abduction, whereas male individuals with FAI primarily adopted a stiffening strategy involving restricted joint motion. Consequently, FAI rehabilitation necessitates sex-specific approaches. Female protocols should emphasize eccentric plantarflexor control and gluteus medius activation training to improve load distribution. Male protocols, while optimizing energy transfer efficiency within the stiffening strategy, must incorporate safeguards against potential focal stress concentration risks (e.g., Achilles tendon). Based on the findings of this study, future research should incorporate sex as a key factor in biomechanical study design and analysis to further elucidate the mechanisms of FAI.

## Data Availability

The original contributions presented in the study are included in the article/supplementary material, further inquiries can be directed to the corresponding authors.
